# Spongiform Venous Malformation Presenting as an Extensive Lesion in the Gluteal Region

**DOI:** 10.7759/cureus.79059

**Published:** 2025-02-15

**Authors:** Ilse Marilú Gutiérrez Villarreal, Circe Ancona Castro, Carlos S Sáenz de León, Génesis A Cabral Rodríguez, Juan Pablo Montemayor Lozano, Roberto Arenas Guzmán

**Affiliations:** 1 Dermatology, Instituto de Seguridad y Servicios Sociales de los Trabajadores del Estado (ISSSTE) Monterrey Regional Hospital, Monterrey, MEX; 2 Infectious Disease, Instituto de Seguridad y Servicios Sociales de los Trabajadores del Estado (ISSSTE) Monterrey Regional Hospital, Monterrey, MEX; 3 Interventional Radiology, Instituto de Seguridad y Servicios Sociales de los Trabajadores del Estado (ISSSTE) Monterrey Regional Hospital, Monterrey, MEX; 4 Mycology, Hospital General Dr. Manuel Gea González, Mexico City, MEX

**Keywords:** magnetic resonance, sclerotherapy, spongiform malformation, vascular malformations, venous malformations

## Abstract

Venous malformations (VMs) are vascular malformations affecting a small portion of the population. A 53-year-old woman presented with a congenital VM on the left gluteal region confirmed through clinical imaging and histopathology. Treatment with sclerotherapy led to significant improvement. VMs often cause pain, swelling, or mobility limitations. Diagnosis relies on Doppler ultrasound and MRI, while sclerotherapy is the preferred treatment due to its safety and efficacy. This case highlights sclerotherapy as a key treatment in a multidisciplinary management approach.

## Introduction

Venous malformations (VMs) are among the frequent types of vascular malformations, occurring in approximately 1 to 5 out of every 10,000 people [1]. VMs often cause pain, swelling, or mobility limitations. Diagnosis relies on doppler ultrasound and MRI, while sclerotherapy is the preferred treatment due to its safety and efficacy [2]. A 53-year-old woman presented with a congenital VM on the left gluteal region, which was confirmed through clinical imaging and histopathology. This case highlights sclerotherapy as a key treatment in a multidisciplinary management approach.

## Case presentation

**Figure 1:**
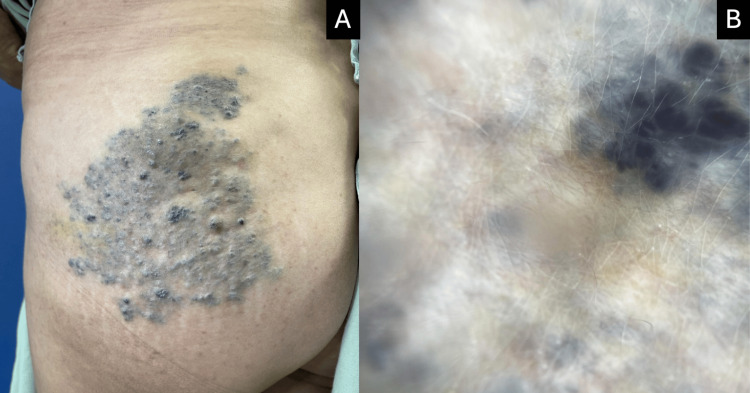
Venous malformation images. (A) Spongiform venous malformation affecting the gluteal region. (B) Dermoscopy shows multiple blue lacunae that tend to group together in a circular arrangement.

A 53-year-old woman with a personal medical history relevant for rheumatoid arthritis since 2015 in control with etanercept and leflunomide presented to us with a 6 cm violaceous neoformation affecting the gluteal region that had been present since birth, with progressive growth of the lesion starting at 18 years of age. Two months prior to the visit, the lesion began bleeding after an unspecified direct trauma, which was the reason for the examination. The patient presented with a dermatosis on the posterolateral aspect of the left lower extremity, extending to the outer gluteal region. It consisted of a vascular-appearing plaque-like neoformation measuring approximately 13×11.5 cm. The lesion was rhomboid in shape, blue-green in color, with well-defined regular borders, an irregular cobblestone-textured surface, and multiple nodules of varying sizes ranging from 5 to 10 mm in diameter (Figure 1A). The lesion was compressible to pressure, and no lesions were found in oral mucosa or annexes. She reported occasional pain in the affected area and slight heaviness of the limb. Dermoscopy showed multiple blue lacunae that tend to group together in a circular arrangement (Figure 1B).

**Figure 2:**
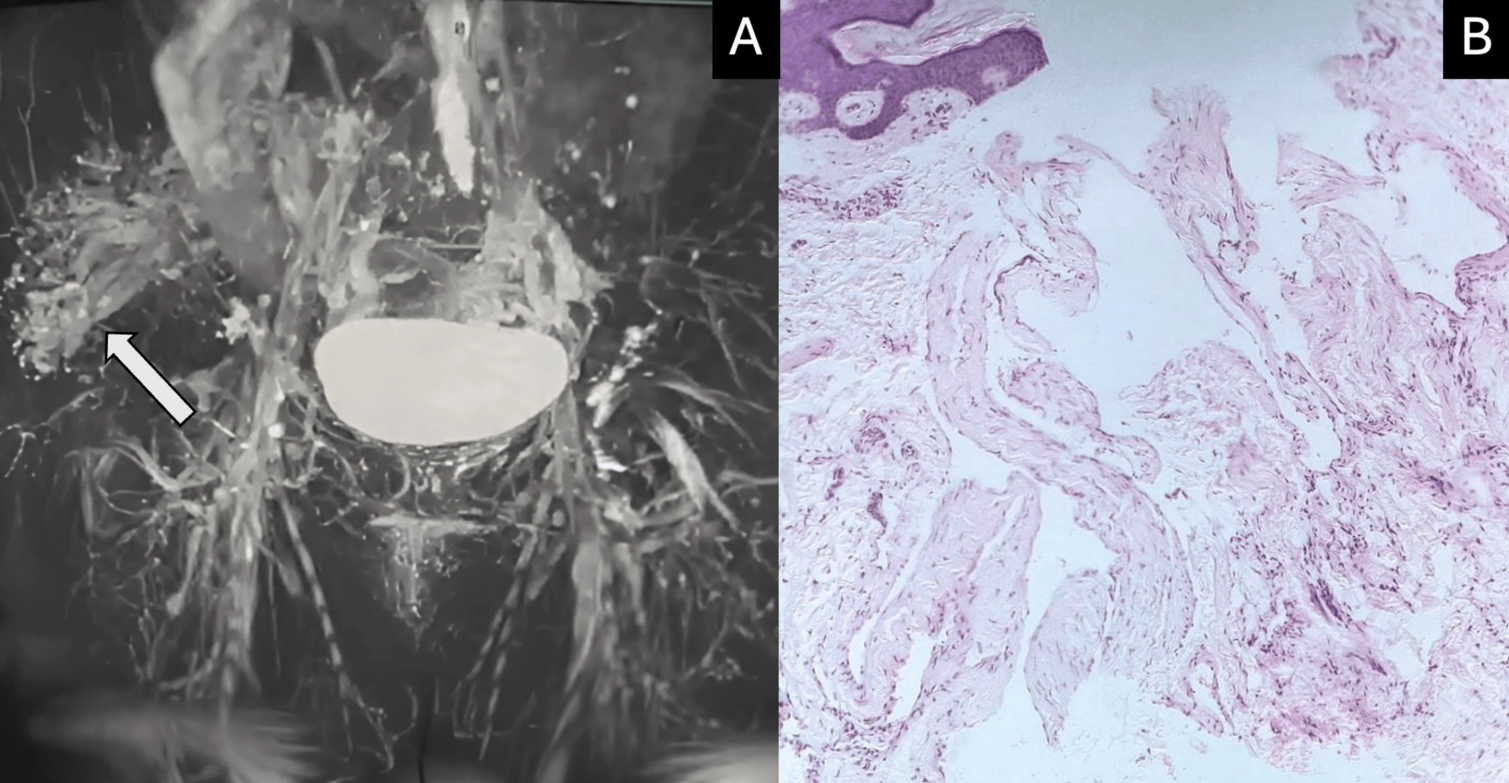
Contrasted MR (A) Shows multiple linear and serpiginous paths showing extension of the lesion marked by an arrow. (B) Dermis with numerous vascular spaces lined by flat endothelial cells and an intercommunicated fibrous stroma.

In our initial approach, a simple ultrasound of the left gluteal region was performed, which revealed an increase in subcutaneous cellular tissue thickness (33 mm) compared to the contralateral side, with heterogeneous, predominantly echogenic characteristics. Doppler ultrasound showed tortuous low-flow vessels and an 8 mm phlebolith. Contrast magnetic resonance imaging of the pelvis was performed, revealing multiple linear and serpiginous paths hyperintense on T2, reported in the subcutaneous cellular tissue of the left gluteal area, affecting the subdermal region with extension to the subcutaneous cellular tissue, gluteus maximus muscle, and the most proximal portion of the iliac crest (Figure 2A). A punch biopsy was performed due to uncertainty regarding the type of vessel involved, which reported an epidermis with loose hyperkeratosis and follicular plugging. In the reticular dermis, numerous vascular spaces lined by flat endothelial cells and an intercommunicated fibrous stroma were observed (Figure 2B). Immunohistochemistry stains were performed, which tested positive for CD34 and negative for podoplanin/D2-40 and CD31.

**Figure 3:**
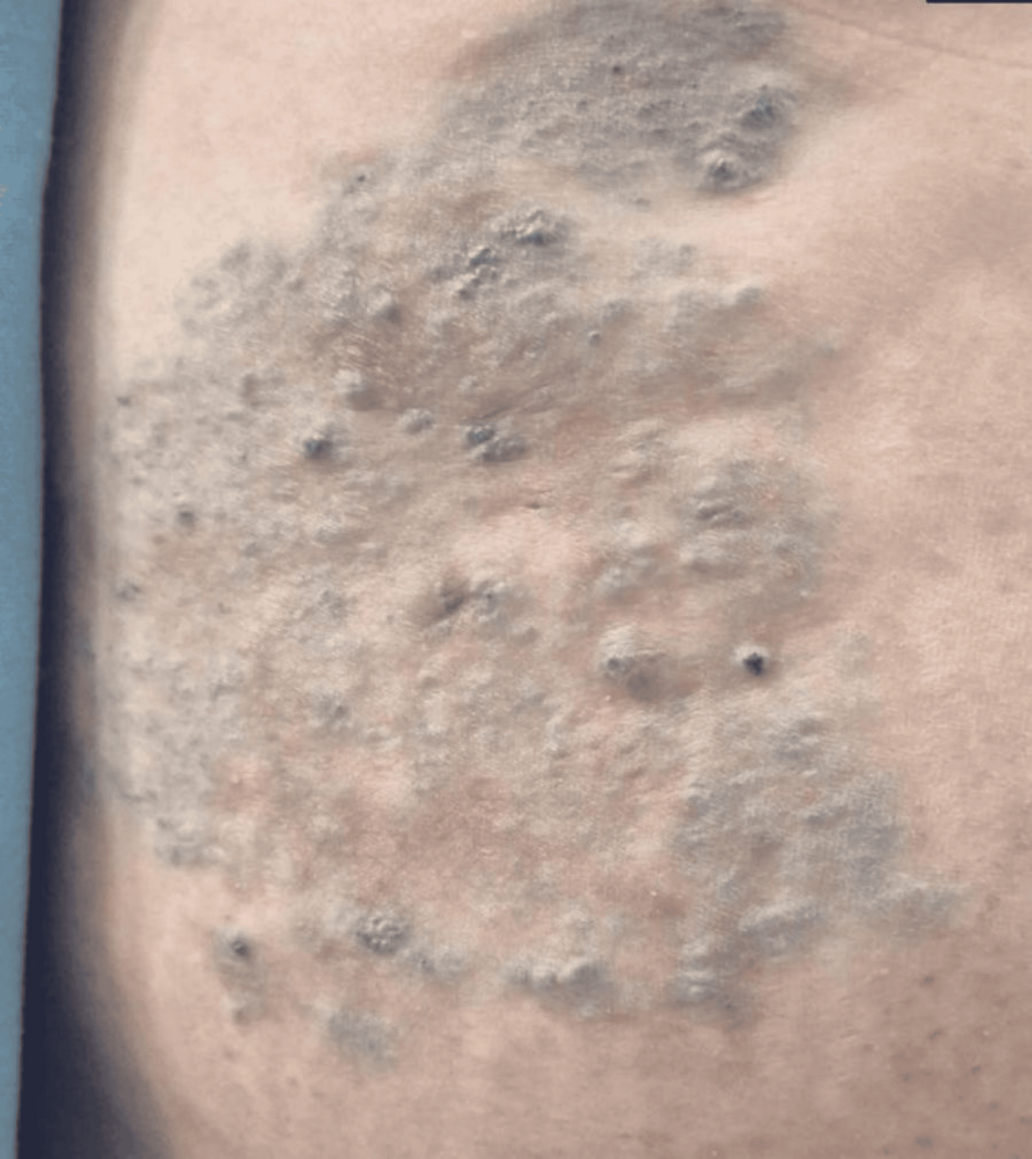
Improvement of the lesion. Improved lesion after one session of 1% polidocanol sclerotherapy.

Considering our diagnostic protocol, the findings suggested a vascular malformation of venous origin, and ultimately, a diagnosis of spongiform venous malformation was made. The patient was treated with a 1% polidocanol sclerotherapy session (with a total volume of 8 mL of the solution injected), achieving significant improvement of the lesion with aesthetic and functional improvement; she did not experience any adverse events (Figure 3).

## Discussion

Venous malformations (VMs) are estimated to occur in approximately 1-5 per 10,000 live births, making them the most prevalent type of vascular malformation (70%) [1]. There is no clear ethnic or gender predisposition, although some studies suggest that venous malformations (VMs) are more frequently observed in females [2,3]. Venous malformations (VMs) are congenital, slow-flow lesions composed of a network of serpentine veins connected by ectatic venous channels that lack vascular smooth muscle [3]. These malformations can be focal, multifocal, or infiltrative and are commonly located in the head and neck (40%), extremities (40%), and trunk (20%) [4].

Pain is the most common symptom associated with venous malformations (VMs). According to Rikihisa et al., pain was most frequently observed in lesions larger than 10 cm (67%), followed by lesions between 5 cm and 10 cm (56%) and those smaller than 5 cm (29%). Subfascial venous malformations (VMs) in the lower extremities are more likely to cause pain. As children age, the frequency of pain increases, reaching 50% by approximately 7 years of age [4,5]. Additional symptoms of venous malformations (VMs) include swelling, varicosity, ulcers, discoloration, itching, and a sense of heaviness, with their manifestation varying based on lesion location, extent, and depth. Despite the movement limitations often reported in venous malformations (VMs) involving muscle tissue [6], the patient in this case did not experience functional or mobility restrictions.

The initial imaging technique at some reference centers for vascular malformations is gray-scale ultrasonography combined with color Doppler imaging and spectral analysis due to its affordability, lack of ionizing radiation, and ability to provide fast, real-time results. However, this method has limitations, such as reduced accuracy in assessing large or deep lesions and difficulties detecting bone involvement. On color Doppler imaging, monophasic venous flow is the typical finding within venous malformations (VMs) [7]. 

Niu et al. reported 74 cases of vascular malformations, with lymphatic malformations being the most common subtype (47 cases) and venous malformations the second most common (15 cases) [8]. Magnetic resonance (MR) imaging is considered the most valuable imaging modality for vascular malformations (VMs) due to its high temporal and contrast resolution, which enables precise evaluation of the malformations and their relationship with adjacent structures. Venous malformations (VMs) typically appear hypointense on T1-weighted images and hyperintense on T2-weighted images and fluid-sensitive sequences. Phleboliths, a characteristic feature of venous malformations (VMs), are visualized as scattered, rounded areas of low signal intensity [8].

Histopathologic diagnosis is rarely necessary. VMs are composed of irregular venous channels lined by flat endothelium, and vascular endothelial cells are negative for lymphatic endothelial markers such as D2-40 and PROX-1. D2-40 binds to a specific marker found in lymphatic endothelial cells, helping to differentiate blood vessels from lymphatic vessels. PROX-1, on the other hand, plays a key role in the differentiation and maturation of lymphatic vessels and is used to evaluate lymphatic malformations and tumors associated with the lymphatic system [3]. Both markers are crucial for studying vascular malformations, but neither was available in our local setting.

There is no fully standardized treatment for venous spongiform malformations (VSMs), and treatment is often sought due to pain or cosmetic concerns. When considering treatment options, factors such as the lesion's pathophysiology, etiology, and potential complications should be considered [9]. The first step in managing vascular malformations is often conservative therapy, particularly when symptoms are mild or intermittent [10].

Treatment options for venous spongiform malformations (VSMs) include surgery, sclerotherapy, and laser therapy, with sclerotherapy being the preferred technique due to its minimally invasive nature, cost-effectiveness, and superior outcomes compared to surgery, which carries a higher risk of intraoperative bleeding and complications [9-11]. Combining these therapies may result in improved outcomes, both in terms of functionality and aesthetics. Well-demarcated, localized lesions tend to respond well to surgical resection [10].

## Conclusions

Venous malformations, particularly when affecting large or extensive areas, often require multiple treatment sessions to address a range of symptoms, from aesthetic concerns to more debilitating issues. Fortunately, sclerotherapy offers a minimally invasive treatment option with low associated risks and generally acceptable outcomes, with many patients opting for repeated sessions. The management of VMs requires a multidisciplinary approach involving a dermatologist, interventional radiologist, vascular surgeon, plastic surgeon, and psychologist to ensure adequate care. Our case highlights the challenges faced by patients with spongiform venous malformations, particularly in obtaining an accurate diagnosis and receiving effective treatment.
